# Potential genotoxic and cytotoxicity of emamectin benzoate in human normal liver cells

**DOI:** 10.18632/oncotarget.18988

**Published:** 2017-07-04

**Authors:** Zhijie Zhang, Xinyu Zhao, Xiaosong Qin

**Affiliations:** ^1^ Clinical Laboratory, Shengjing Hospital, China Medical University, Shenyang 110011, China; ^2^ Complex Laboratory, College of Laboratory Medicine, Dalian Medical University, Dalian 116044, China

**Keywords:** emamectin benzoate, human liver cells, potential genotoxic, cytotoxic effects, mitochondrial-mediated apoptotic

## Abstract

Pesticide residue inducing cancer-related health problems draw people more attention recently. Emamectin benzoate (EMB) has been widely used in agriculture around the world based on its specificity targets. Although potential risk and the molecular mechanism of EMB toxicity to human liver has not been well-characterized. Unlike well-reported toxicity upon central nervous system, potential genotoxic and cytotoxicity of EMB in human liver cell was ignored and very limited. In this study, we identify genotoxicity and cytotoxicity of EMB to human normal liver cells (QSG7701 cell line) *in vitro*. We demonstrate that EMB inhibited the viability of QSG7701 cells and induced the DNA damage. Established assays of cytotoxicity were performed to characterize the mechanism of EMB toxicity on QSG7701 cells. Typical chromatin condensation and DNA fragmentation indicated the apoptosis of QSG7701 cells induced by EMB. And the intracellular biochemical results demonstrated that EMB-enhanced apoptosis of QSG7701 cells concurrent with generated ROS, a loss of mitochondrial membrane potential, the cytochrome-c release, up regulate the Bax/Bcl-2 and the activation of caspase-9/-3. Our results of EMB induces the death of QSG7701 cells maybe via mitochondrial-mediated intrinsic apoptotic pathways would contribute to promote the awareness of EMB as an extensive used pesticide to human being effects and reveal the underlying mechanisms of potential genotoxic.

## INTRODUCTION

Pesticide residue inducing cancer-related health problems draw people more attention recently [1, 2]. Emamectin benzoate (EMB), an avermectin derivative, is found to be effective against variety of pests and now widely used [3]. This function of EMB has been ascribed to its neurotoxicity via stimulating γ-aminobutyric acid (GABA) receptor and glutamate-gated chloride channels [4]. Initially, EMB is considered to be safe and less toxic because GABA-reactive neurons are limited in central nervous system in human beings [5]. However, EMB is lipophilic and easily pass through cell membrane into cytoplasm [6]. The United States Environmental Protection Agency (EPA) Pesticide Fact Sheet classifies EMB as an extremely toxic compound for mammals [7]. And more and more evidences have illustrated that long-term accumulation of anthropogenic origin chemical substances poses potential risk in human health.

EMB is still unknown of its latent carcinogenic risk while avermectin has been reported of its significant genotoxic and cytotoxicity. Genotoxic compound and its metabolites could cause DNA damage via breaking DNA single-strand or double-strand [8]. Under physiological conditions or limited DNA damage, Poly (ADP-ribose) polymerase (PARP) plays an important role in protecting organism health in cell death program by over-activation or participating extracellular signal regulated cascade [9, 10]. Apoptosis is a fundamental cellular event during development and is critical for the cytotoxicity induced by drugs characterized by the cleavage of chromatin DNA into inter-nucleosomal fragments [11–13]. Apoptosis can be initiated through one of two pathways, intrinsic pathway of triggering apoptosis is induced by intracellular signals such as DNA damage and mediated by releasing death factors into cytosol from mitochondrial and regulation of Bcl-2 family proteins, while extrinsic pathway is mediated by death receptors [14, 15]. Both pathways induce cell death by activating caspases, which then kill cell by degrading key structural and nuclear proteins [16].

Recently, EMB has been identified having adverse effects on multiple non-target organisms, such as male rat hepatocytes [17, 18]. However, detailed cytotoxic effects and molecular mechanisms underlying EMB-mediated human liver cells genotoxic effects remain to be further elucidated. Human liver is primarily responsible for the metabolism of toxic substances, meanwhile contamination vegetables and fruits are main source of pesticide residue. In the current study, QSG7701 cells were used as a model for typical human normal liver cells to evaluate the toxicological effects of EMB for human liver. Our study demonstrated that EMB-enhanced apoptosis of QSG7701 cells concurrent with DNA damage, a loss of mitochondrial membrane potential, the cytochrome-c release, up regulate the Bax/Bcl-2 and the activation of caspase-9/-3. It shows that EMB has potential genotoxicity and significant cytotoxicity to human liver cells.

## RESULTS

### Viability inhibition of QSG7701 cells by EMB

**Figure 1:**
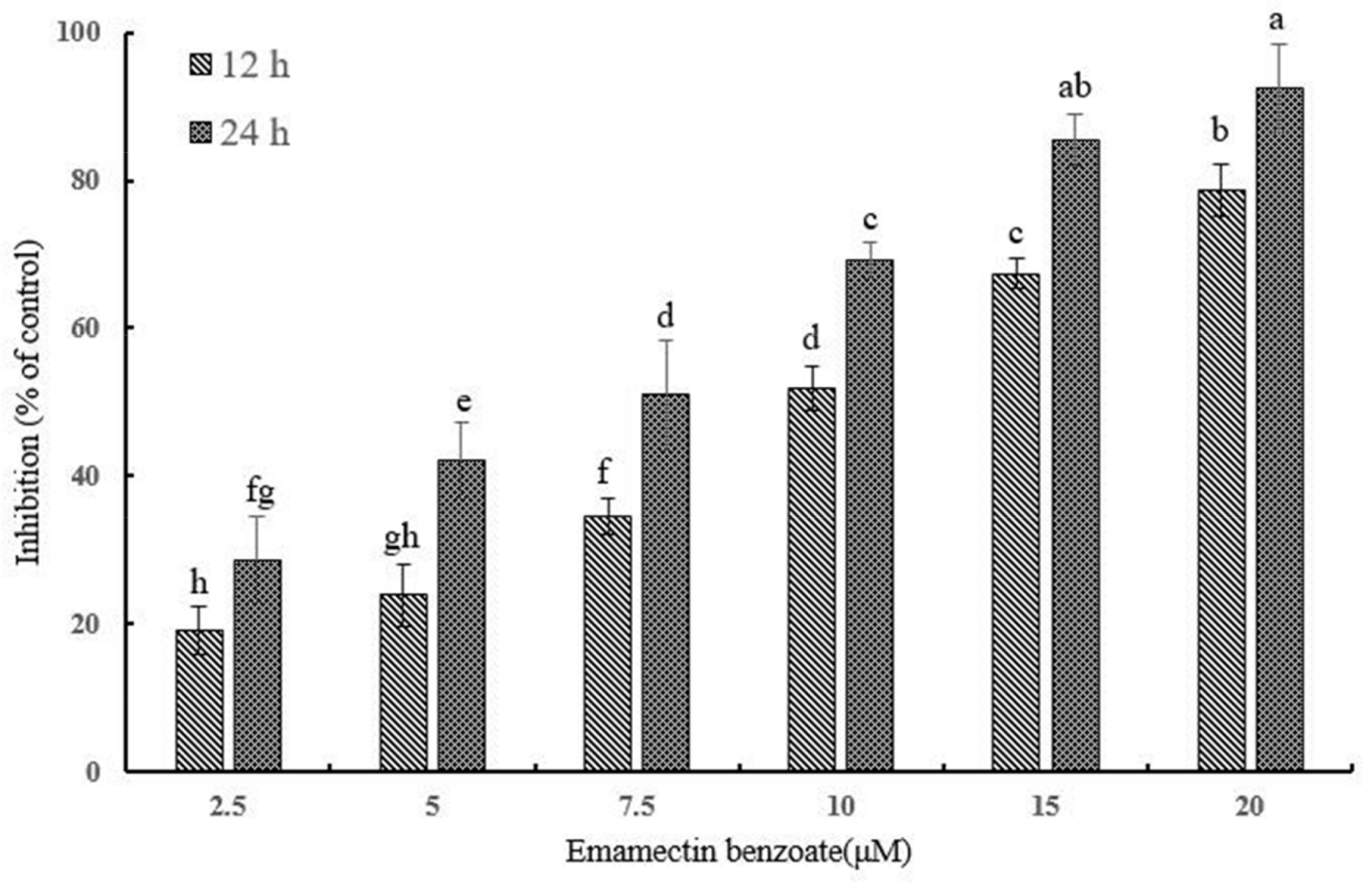
Cytotoxicity of EMB on QSG7701 cells Cell viability of QSG7701 cells treated with 2.5, 5, 7.5, 10, 15 and 20 μM EMB for 12 and 24 h. The data are shown as a percentage of the control group and the means ± SD of three independent experiments. Different small letters indicate significant differences (P≤0.05) between any two groups.

In order to access the cytotoxic influence that EMB can exert on QSG7701 cells, we performed the MTT assay. As shown in Figure 1, with the increasing concentrations of EMB, the viability of QSG7701 became worse and worse. The proliferation inhibition rates of QSG7701 cells were 19.12 ± 3.32%, 23.87 ± 4.22%, 34.57 ± 2.46%, 51.91±2.99%, 67.42±2.16% and 78.67±3.59% after 12 h of treatment with EMB at the concentrations of 2.5, 5, 7.5, 10, 15 and 20 μM, which finally reached to 28.71±5.86%, 42.21±5.11%, 50.98±7.49%, 69.18±2.53%, 85.38±3.51% and 92.52±5.98% after 24 h treatments. IC_50_ values for EMB treatments of 12 and 24 h are given in Table 1. The date were 9.98 and 5.71 μM respectively.

**Table 1:** The half maximal inhibitory concentration (IC_50_) of QSG7701 cells exposed to EMB

Cell lines	Time(h)	IC_50_(μM)	Toxicity regression equation (y=)	95%(confidence limits)	
				Lower	Upper
QSG7701	12	9.98 a	1.72x + 3.27	8.39	11.91
	24	5.71 b	2.03x + 3.48	4.96	6.55

Small alphabet indicated statistically significant differences between two groups in the same column (P≤0.05).

### EMB induces DNA damage

**Figure 2:**
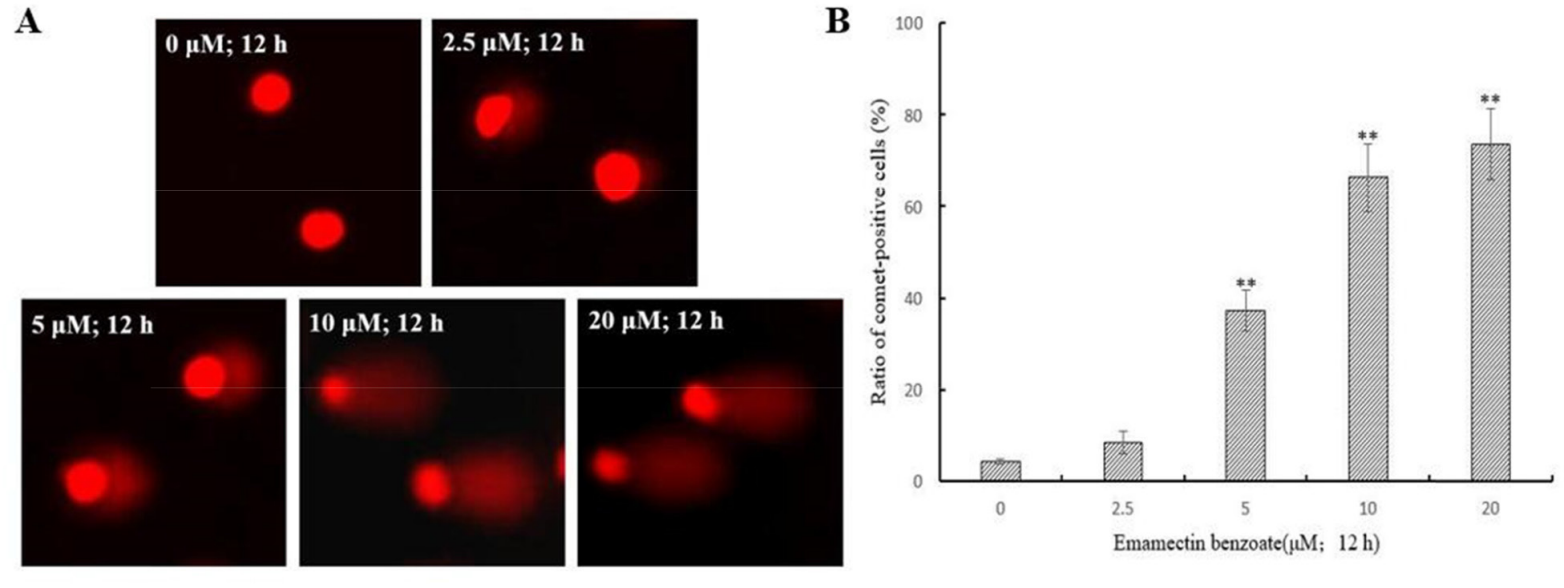
EMB induced DNA damage in QSG7701 cells DNA fragments were shown as comet images in alkaline gel electrophoresis (200 ×) **(A)**. Percentage of comet-positive cells in the treatment of EMB for 12 h analysis results were shown in the right panel **(B)**. The data are shown as the means ± SD of three independent experiments. **P < 0.01.

In order to know if EMB cause DNA damage, the alkaline comet assay was used since the migrated DNA fragments can form a comet-like image [13]. In the control, comet heads were concentrated with high-density DNA that accompanied with smooth margin and intact nuclei, and the frequencies of comet cells were ∼5%. In the EMB treatments, the fluorescence intensities of the comet heads were weaker than in the controls, and the damaged DNA migrated out of the heads and formed broom-shaped tails (Figure 2A). The percentages of comet-positive QSG7701 cells showed significantly increase in a dose-dependent manner after 12 h of exposure to EMB (Figure 2B). The distributions of QSG7701 cells exposed to EMB including the tail DNA, tail lengths and tail moment are presented in Table 2. These results demonstrated that EMB induced the DNA damage of QSG7701 cells in a dose-dependent manner.

**Table 2:** The parameters of DNA damage in alkaline comet assay with QSG7701 cells that were exposed to the different concentrations of EMB in 12 h

Concentrations (μM)	Comet assay parameters		
	Tail DNA (%)	Tail length (μm)	Tail moment
0	0.83 ± 0.07 e	2.18 ± 0.89 e	0.12 ± 0.04 d
2.5	14.22 ± 3.69 d	8.68 ± 0.77 d	9.31 ± 1.74 d
5	32.98 ± 4.08 c	20.85 ± 3.09 c	30.01 ± 7.79 c
10	46.99 ± 5.21 b	36.37 ± 1.31 b	55.58 ± 6.39 b
20	69.42 ± 2.87 a	43.79 ± 0.76 a	64.39 ± 4.28 a

Data represent mean values (±SD) of three independent experiments.

Different small letters in the same column indicate significant differences (P≤0.05) between any two groups.

### γH2AX foci revealing DNA double-strand breaks induced by EMB

**Figure 3:**
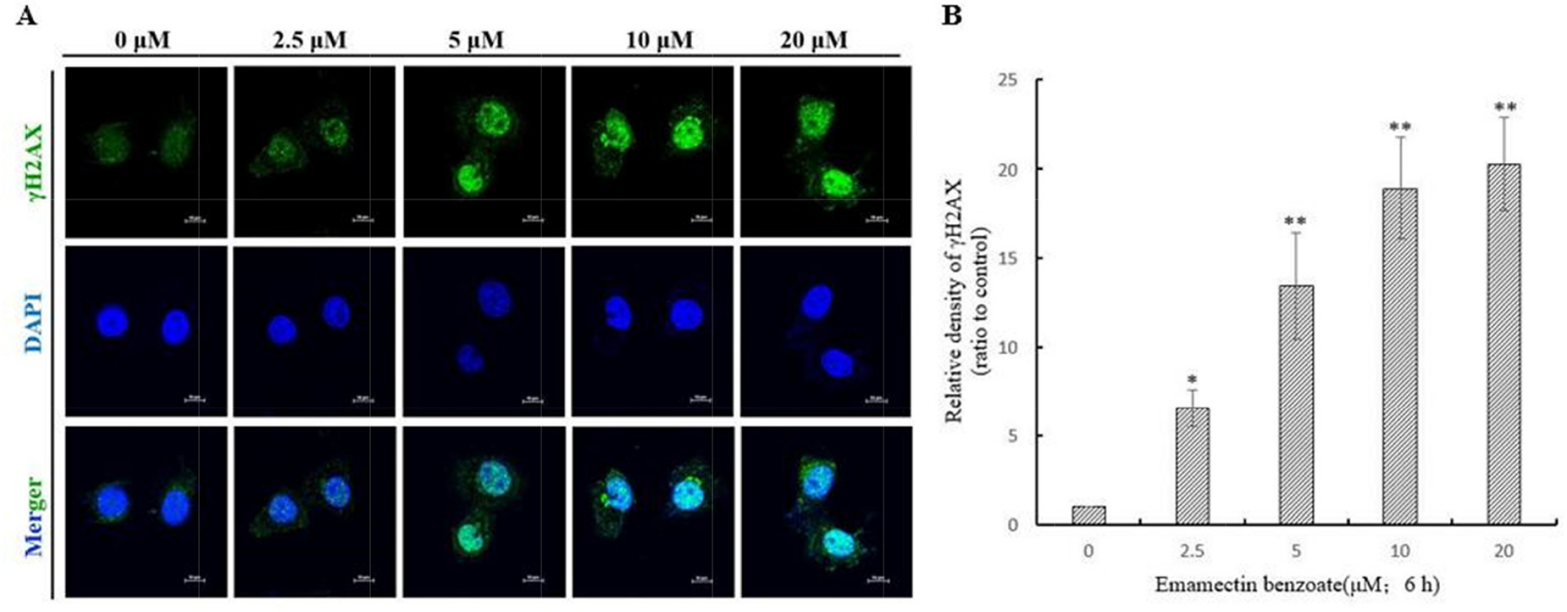
DNA double strand breaks were illustrated by γH2AX foci formation in QSG7701 cells γH2AX foci formation images of QSG7701 cells in the treatment of 2.5, 5, 10, 20 μM EMB for 6 h **(A)**. Anti-γH2AX monoclonal antibody was used to detect DNA damage foci immunofluorescence and DAPI was for nuclei staining. The relative density of γH2AX in the treatment of EMB for 6 h analysis results were shown in the right panel **(B)**. Data were represented as means ± SD from three independent experiments. *P ≤ 0.05 and **P < 0.01.

The immunofluorescent images of γH2AX-stained QSG7701 cells are shown in Figure 3A. It shows that EMB induced a dose-dependent induction of γH2AX foci in QSG7701 cells. In the control, QSG7701 cells had little γH2AX foci in the nuclei. All the treatments with EMB induced foci formation, with the increased number of γH2AX in nuclei. The data in Figure 3B shows that EMB exhibited significant effects (p < 0.01) on γH2AX foci formation in QSG7701 cells in a dose-dependent manner.

### EMB induced apoptosis in QSG7701 cells

**Figure 4:**
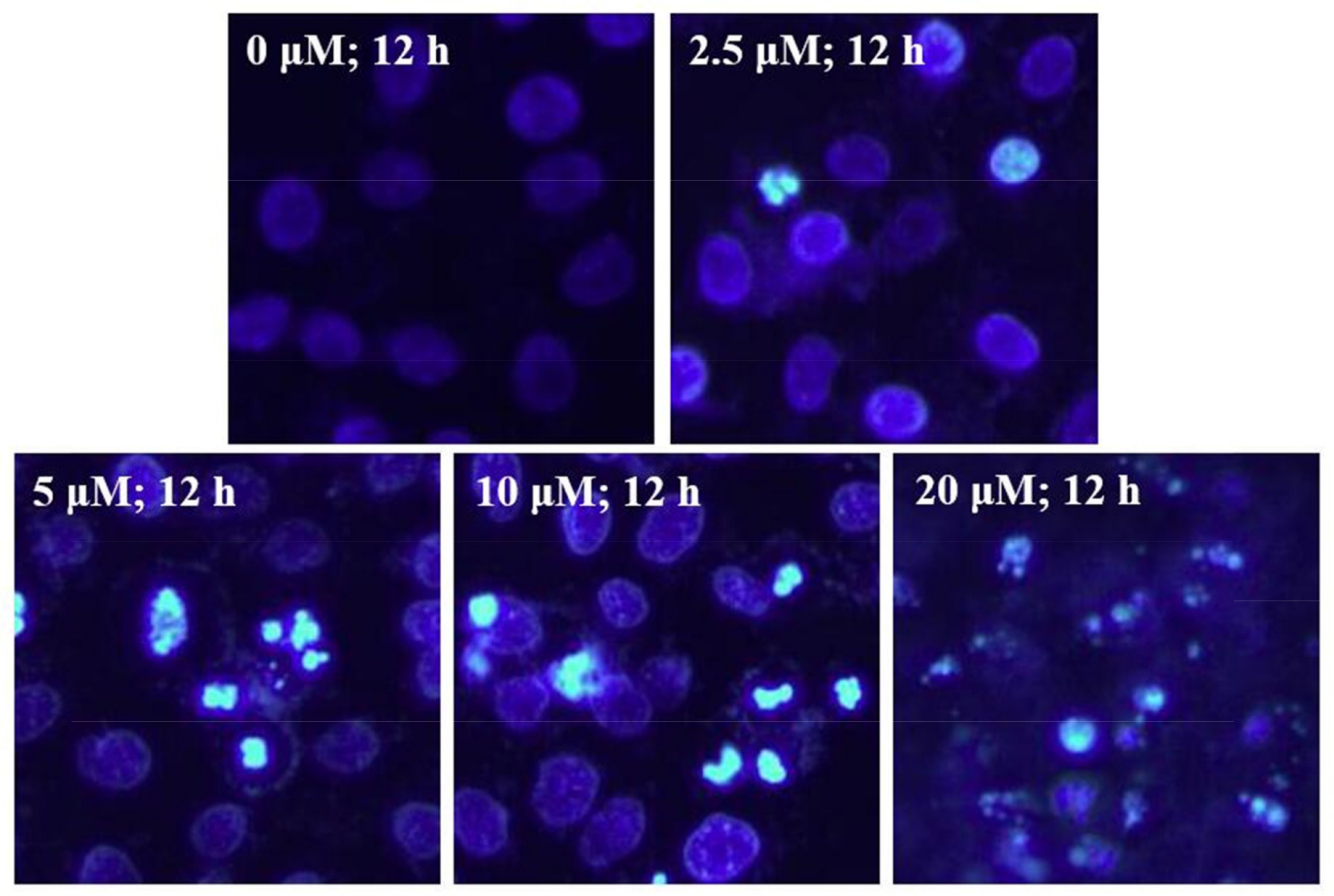
EMB-induced chromatin condensation in QSG7701 cells Cell nuclei were stained with Hoechst 33258 and observed by fluorescence microscopy. Representative images of apoptotic chromatin condensation were treated with EMB.

**Figure 5:**
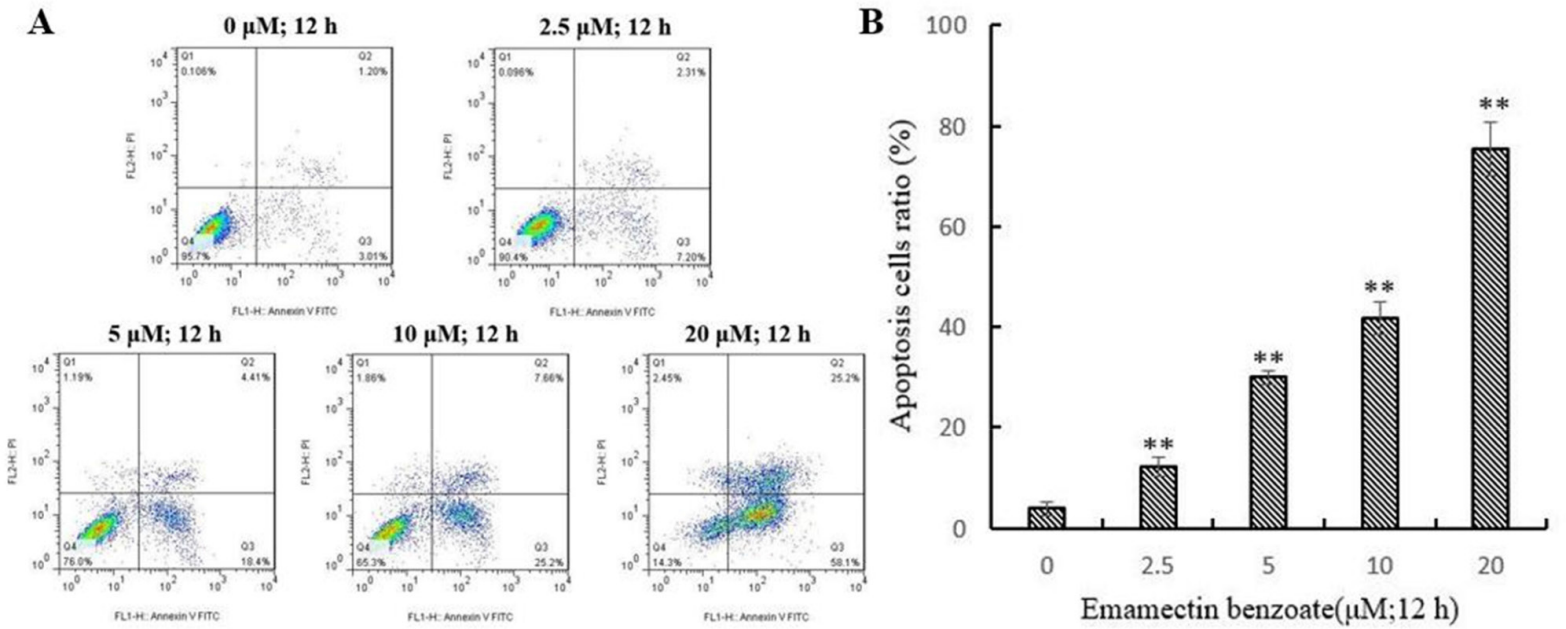
EMB-induced apoptosis in QSG-7701 cells Representative flow cytometric analysis for cells stained with Anexin V-PI following treatment with 2.5, 5, 10, 20 μM EMB and 0.1% DMSO was used as a control for 12 h **(A)**. The lower right panel shows the early apoptotic cells and the upper right panel shows the late apoptotic cells or undergoing necrotic cells. Quantitative data was shown in the right panel **(B)**. The data represent the means ± SD values of three experiments in triplicate. **P ≤ 0.01.

After treatment with 2.5, 5, 10 and 20μM of EMB for 12 h, a growing number of cell had started to chromatin condensation and undergo fragmentation (Figure 4). Flow cytometry method was then used to quantify the effect of EMB on QSG7701 cells (Figure 5A). The flow cytometry assay demonstrated that the ratio of apoptotic cells increased from 3.83 ± 1.41% in untreated cells to 12.28 ± 1.87%, 29.89 ± 1.46%, 41.86 ± 3.23% and 75.41 ± 5.37% in 2.5, 5, 10 and 20μM EMB-treated cells, respectively, which demonstrated that EMB increased the apoptotic of QSG7701 cells in a dose-dependent manner (Figure 5B).

### EMB induces mitochondrial dysfunction

**Figure 6:**
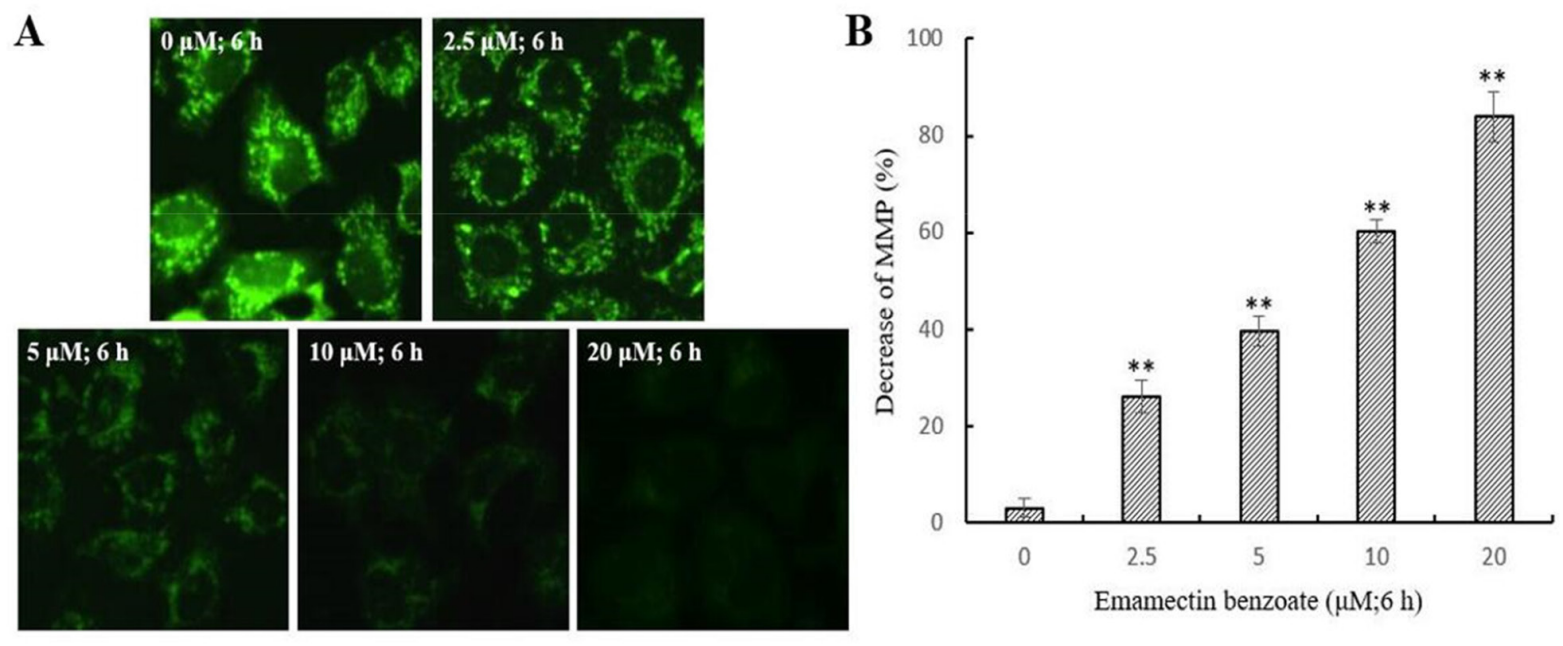
Depolarization of *ΔΨm* in EMB-treated QSG7701 cells QSG7701 cells were pre-treated with 2.5, 5, 10 and 20 μM of EMB for 6 h and then loaded with Rh-123. The quantification of Rh-123 accumulation in mitochondria (green fluorescence) was analyzed by flow microscopy (200×) **(A)**. Data was shown in the right panel **(B)**. Data are the means ± SD of three independent experiments and expressed as the percentage of the control cells. **P <0.01.

A reduction in *ΔΨm* may trigger apoptosis, and it is usually considered as an earlier event before cytochrome and other mitochondrial factors in the apoptosis cascade [22]. Therefore, we assessed the effect of EMB on *ΔΨm* in QSG7701 cell lines. Fluorescent microscopy observation of QSG7701 cells exposed to 2.5, 5, 10 and 20 μM EMB for 6 h demonstrated they had gradually decreased green fluorescence intensity in the mitochondria (Figure 6). The results indicated that the reduction of *ΔΨm* in QSG7701 cells was caused by EMB in a dose-dependent manner.

### Effects of EMB on the expression and activity level of apoptosis-related proteins in QSG7701 cells

**Figure 7:**
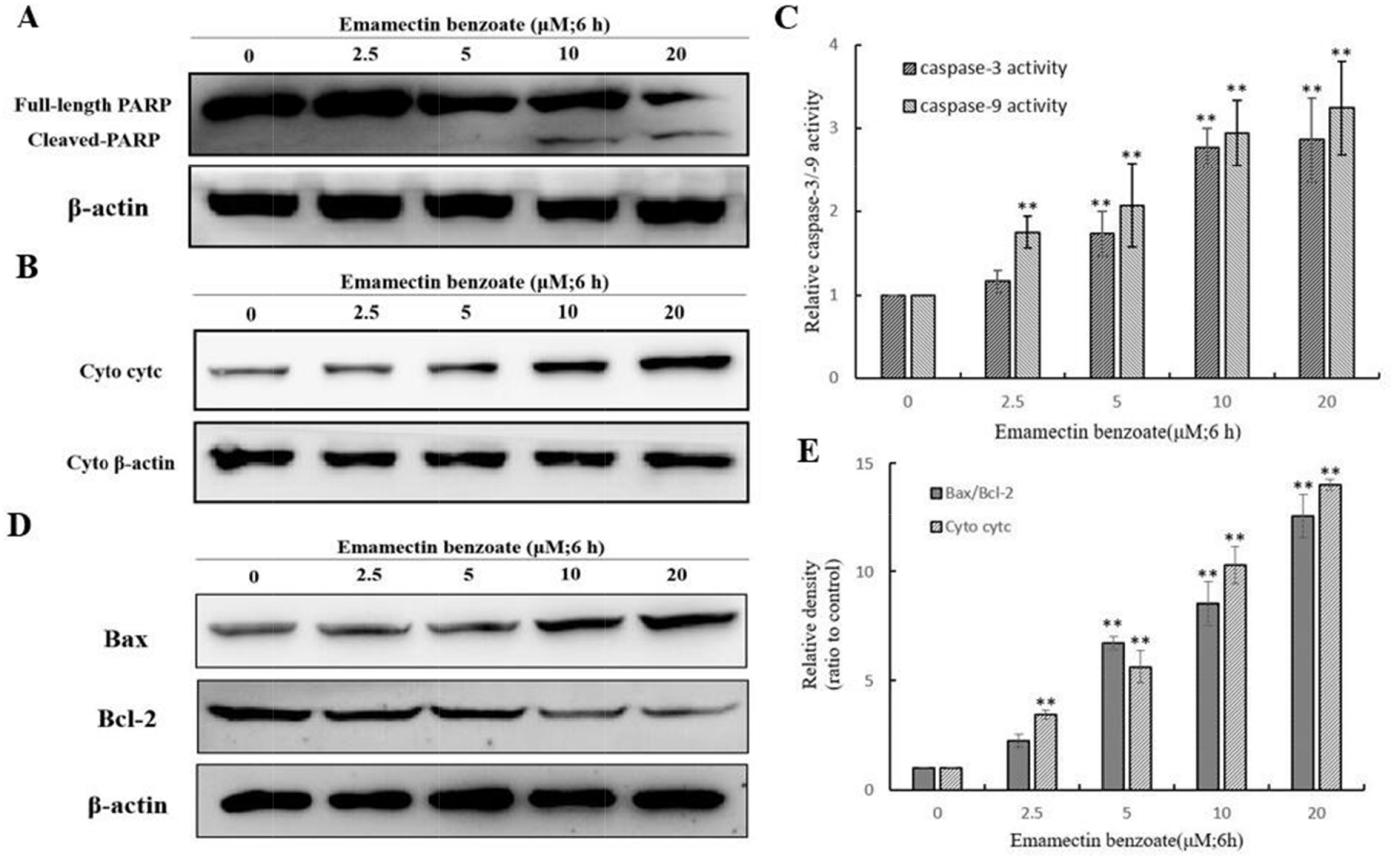
Effect of EMB on the expression of apoptosis-related proteins in QSG7701 cells Cells were fractionized following treatment with 2.5, 5, 10, 20 μM EMB for 6 h and PARP was detected by Western blot analysis **(A)**. Markers of mitochondrial pathway related proteins as cytc, Bax and Bcl-2 were detected by Western blot analysis **(B, D)**. The activation of caspase-3 and caspase-9 were shown in right panel **(C)**. Cyto denote cytosolic fractions. β-actin was used as an equal loading control. Data were represented as means ± SD from three independent experiments. **P < 0.01.

To elaborate the reaction mechanism of EMB-triggered apoptosis, we tested the constituent of apoptosis-associated proteins in QSG7701 cells after the EMB processing under various concentrations (0, 2.5, 5, 10 and 20 μM) for indicated times. PARP cleavage in the EMB treatment than control group assured that the apoptosis induced by the EMB treatment involved DNA fragmentation which was related to PARP cleavage (Figure 7A).

In order to examine the release of cytochrome-c into the cytosol, extracted cytosolic fractions of QSG7701 cells treated with EMB were analyzed by Western blot assay. As shown in Figure 7B, cytochrome-c release was increased in a dose-dependent manner in EMB-induced QSG7701 cells.

Meanwhile, in QSG7701 cells with EMB-induced 6 h, the expression level of pro-apoptotic protein bax was elevated and anti-apoptosis protein Bcl-2 was down-regulated in a dose-dependent manner (Figure 7D). The band intensity histogram in Figure 7E showed the significant increase of cyt-c, Bax/Bcl-2 in a dose-dependent manner after EMB treatment.

### Effects of EMB on caspase-3/-9 activation in QSG7701 cells

Caspases are a family of cysteine proteases which are involved in apoptosis modulation. In pro-apoptotic stage, caspases response the signals and propagate apoptosis, cleavage of caspase 9 and caspase 3 to its active form considered as very important event in mitochondrial apoptosis pathway [23]. The EMB-treated QSG7701 cells were investigated for the activation of caspase-9/-3 by colorimetric enzymatic assay. As shown in Figure 7C, both activation of caspase-9 and caspase-3 increased when the cells were treated with higher concentrations of EMB. Our results suggested that the EMB-induced apoptosis was mediated through the activation of caspase-9/-3.

### EMB induced generation of intracellular ROS in QSG7701 cells

**Figure 8:**
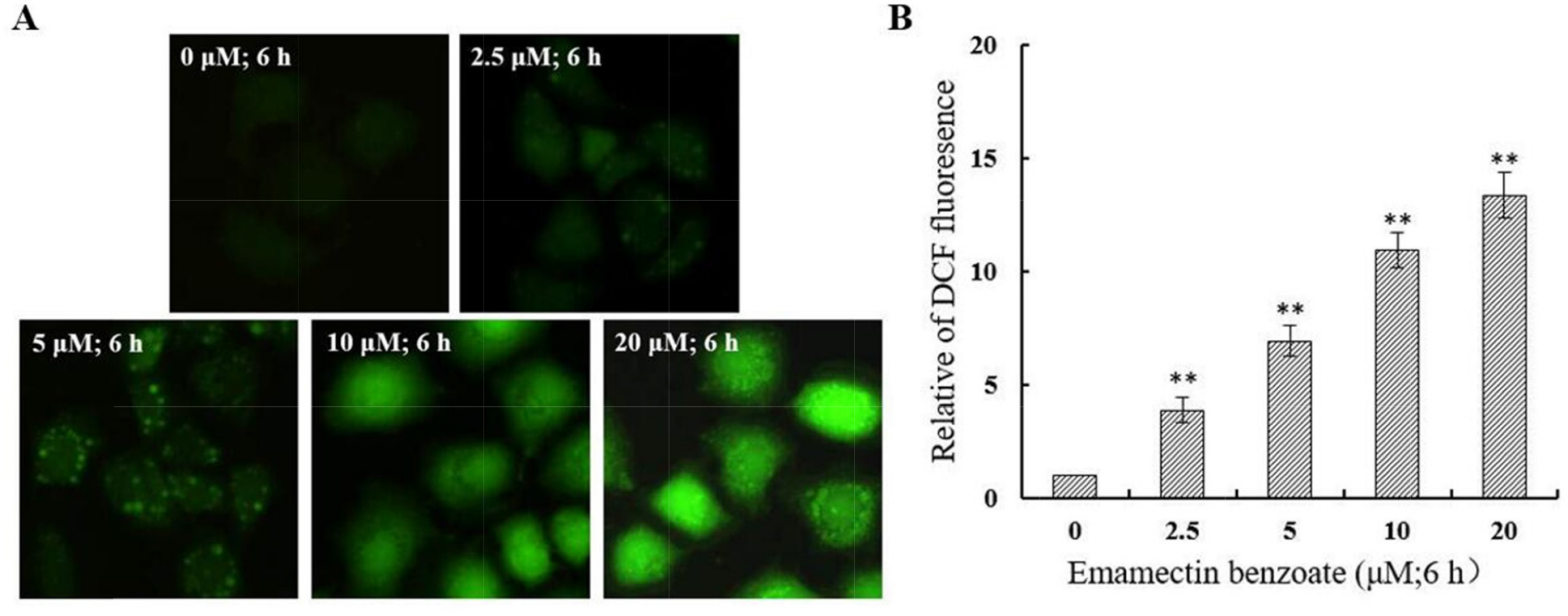
EMB induced generation of intracellular ROS in QSG7701 cells The treated cells were collected for DCFH-DA fluorescence was examined by fluorescent microscopy (200×) **(A)**. The data of ROS generation was shown in the right panel **(B)**. The data are shown as the means ± SD of three independent experiments. **P ≤ 0.01.

We analyzed the intracellular ROS level using fluorescence microscopy after DCFH-DA being hatched in cells. In comparison to 0μM EMB group, the intracellular ROS of QSG7701 cells which were stained by DCFH-DA have dramatically raised in amount after 2.5, 5, 10 and 20 μM EMB treatment (Figure 8). The studies suggested ROS increases in a dose-dependent way in EMB-treated QSG7701 cells.

## DISCUSSION

EMB has been reported to maintain acute and longer-term toxicity (0.075 mg/kg for mice, oral) and cytotoxic effects of different types of mammalian cells across a widely spread of concentrations [7, 24, 25]. In this study, we have identified the EMB as a potential genotoxic compound corresponding to significant genotoxicity and cytotoxicity to human normal liver cells *in vitro*. We demonstrated that the underlying mechanism of EMB effects relies on mitochondrial apoptotic pathway. EMB is already extensively used as a broad spectrum anthelmintic drug with few cytotoxic effect observed on human liver cells. To assess its cytotoxic effect, we primarily tested the EMB against QSG7701 cells effect by MTT assay. It identified time- and concentration- dependent manner of EMB inhibiting viability of QSG7701 cells, which demonstrates that EMB has cytotoxicity to QSG7701 cells (Figure 1).

Over the past years, genotoxicity of agrochemicals is becoming more serious, the potential mutagenic and carcinogenic to exposed organisms when reacts with nuclear DNA is one of the most serious side effects on human beings [26]. Genotoxicity of EMB to human beings especially human liver cells has rarely been reported and underlying mechanism needs to be further illustrated. The alkaline comet assay, also known as single-cell gel electrophoresis, was elected to identify DNA fragments for it’s highly sensitive [27]. DNA damage caused by DNA strand breaks and pesticide cleave can be detected by this sensitive fluorescent microscope-based method. Figure 2 shows the size of comet tails increase which is distinct, denatured or cleaved DNA fragments migrating out of the nucleus caused by gel electrophoresis, while head size decreases upon different concentration of EMB. Therefore, with increasing concentrations of EMB, QSG7701 cells were observed to represent extent DNA strand breaks liberated from the heads of comets using the comet assay under alkaline conditions. Besides, DNA damages such as DNA double strand breaks induced by replication stress, ionizing radiation, exogenous stress and drugs can also be detected by another sensitive mark called γH2AX, which is phosphorylation product of histone H2AX [28]. γH2AX aggregates in nuclei and with ≥ 4 γH2AX foci per cell, the damage of DNA can be determined (Figure 3). It provides a sensitive and valuable technique to detect and determine DNA damage caused by EMB. Figure 3B depicted that EMB can induce concentration-dependent formation of γH2AX which means EMB induced DNA double-strand breaks in QSG7701 cells. In present study, we have identified the DNA stand breaks through presence of γH2AX foci via comet assay. The outcome suggests that EMB may be a potently genotoxic medium that leads to human liver cellular DNA damage *in vitro*.

The main characteristic morphological sings of apoptosis are DNA fragments and condensed chromosomal DNA [29]. Our further study verified whether EMB inhibits QSG7701 cells viability by inducing cell apoptosis. These preliminary observations of condensed chromosomal DNA and fragmented nuclei (Figure 4) suggested that EMB induced QSG7701 cells damage through apoptotic pathways. Meanwhile, Flow cytometry results indicated increasing number of QSG7701 cells apoptosis upon different concentrations of EMB (Figure 5).

Apoptosis can be initiated through one of the two pathways, the intrinsic pathway is mediated by mitochondrial and extrinsic pathway is mediated by cell death receptor. The disruption of *ΔΨm* and cytochrome-c release are the main characteristic of intrinsic pathway. Apoptotic proteins that target mitochondria may cause mitochondrial swelling through increase the permeability of mitochondrial membrane and cause apoptotic effectors to leak out. Thus, the disruption of the *ΔΨm* is recognized as key point in apoptotic cascade and it emerged in EMB inducing apoptosis of QSG7701 cells in a dose dependent manner (Figure 6). Cytochrome-c is also released from mitochondria into cytosol due to reduction of *ΔΨm* [30], and serves as a initiate caspase activator in the process of apoptosis anchored in the inner membrane of mitochondria [31]. We identified the release of cytochrome-c from mitochondria in EMB-treated QSG7701 cells via Western blotting (Figure 7). The underlying mechanisms of EMB induced cell death of QSG7701 cells has been demonstrated as a mitochondrial-dependent intrinsic pathway of apoptosis regarding significant *ΔΨm* collapse and cytochrome-c release. Meanwhile, the rise of intracellular reactive oxygen species (ROS) generation through the mitochondrial dysfunction resulted in *ΔΨm* collapse and a release of cytochrome-c. The increased production of ROS by EMB may lead to the initiation of apoptosis in QSG7701 cells.

Caspases play the central role in transduction of intrinsic apoptotic signals, including two types of proteins: initiator caspases and effector caspases. Initiator caspases begin as inactive precursors, but upon receiving an apoptotic signal such as binding to specific oligomeric activator protein, proteolytic process to generate active enzyme then undergoes [19, 32]. Among them, caspase-3 is the major effector protein of apoptosis that is activated by an initiator caspase as caspase-9. In current study, both caspase-3 and caspase-9 were activated (Figure 7C) suggesting EMB-induced QSG7701 cell apoptosis may rely on activated caspases to degrade intracellular proteins to carry out cell death program.

Bcl-2 family members are considered to regulate apoptosis by controlling the formation of mitochondrial apoptosis- induced channel, an ion channel formed on outer mitochondrial membrane response to certain apoptotic stimuli, and related to the cytochrome c release [14]. The anti-apoptotic protein Bcl-2 could suppresses the release of cytochrome c and induces its redistribution to the cytosol, and also inhibit the pro-apoptotic protein Bax [33, 34]. These molecules cause activation of caspase-9/3 contribute to subsequently cell death [35]. In our study, the increase ratio of Bax/Bcl-2 (Figure 7B) caused by EMB results in the release of cytochrome-c which activates caspase-9 and caspase-3, finally leads to death of QSG7701 cells.

In summary, our study provided results pertaining to new information on EMB, which formally regarded as a substance of high environmental safety based on its specificity targets, has the ability to induce the death of human liver cells. Unlike well-reported toxicity upon central nervous system, potential cytotoxicity and genotoxic through long-term accumulation in non-nervous cell was ignored and limited. Our results of EMB caused the death of QSG7701 cells maybe via mitochondrial-mediated intrinsic apoptotic pathways would contribute to promote the awareness of EMB as a widely used pesticide to human health effects and reveal the underlying mechanisms of potential genotoxic. However, our current results cannot demonstrate the induction of apoptosis is directly related to the genotoxicity and the detailed targets on liver cells induced by EMB which will be our focus in future studies.

## MATERIALS AND METHODS

### Chemicals and reagents

Emamectin benzoate (98% purity, EMB) was purchased from Sigma-Aldrich Canada. The following were obtained from Sigma–Aldrich (St. Louis, MO, USA): dimethyl sulfoxide (DMSO), phosphate-buffered saline (PBS, pH 7.4), 3-(4, 5-dimethylthiazol-2-yl)-2, 5-diphenyl-tetrazolium bromide(MTT), 2′, 7′-Dichlorodihydrofluorescein diacetate (DCFH-DA), phenylmethanesulfonyl fluoride (PMSF), bovine serum albumin (BSA), Rhodamine123 (Rh-123), RIPA lysis buffer. All antibodies were obtained from Cell Signaling Technology (Beverly, MA, USA).

### Cell culture

The human normal liver cell line QSG7701 was obtained from the Cell Bank of Type Culture Collection of Chinese Academy of Sciences, Shanghai Institutes for Biological Sciences, Chinese Academy of Sciences (Shanghai, China). It was cultured in DMEM medium with 10% FBS and 1% penicillin-streptomycin solution (Gibco, Grand Island, NY, USA), and incubated at 37 °C in a humidified 5% CO_2_ atmosphere incubator.

### Cell viability assay

Cell viability, as an testing endpoint of cytotoxicity, was determined by MTT assays [19]. This assay measures the conversion of MTT to purple formazan by succinate dehydrogenase of the intact mitochondria of living cells. QSG7701 cells were seeded into 96-well plates (150 μL/well) at a density of 1×10^5^ cells per milliliter in DMEM (10% FBS) incubated for 24 h and then threated with 2.5, 5, 7.5, 10, 15, and 20 μM EMB. Fresh medium containing 0.1% DMSO was used as negative control. After 12 and 24 h of treatment, 20 μL MTT (5 mg/mL) was added to each well. After incubation for 4 h at 37°C, the supernatants of culture media was aspirated and 150 μL DMSO was added to each well to dissolve the formazan crystals. Then, the reading of absorbance was taken at 572 nm by a Synergy H1 microplate reader (Bio-Teck, Winooski, VT, USA).

### Alkaline comet assay

After the QSG7701 cells were treated by 2.5, 5, 10 and 20 μM EMB for 12 h, 40 μl aliquots of cell suspensions (1 × 10^4^ cells) were mixed in 120 μL of 1% low-melting agarose at 37 °C. The agarose was curdled at 4 °C for 10 minutes and the slides were immersed in fresh lysis solution (10% DMSO, 1% Triton X-100, 10 mM Tris–HCl, 100 mM EDTA, 2.5 M NaCl, pH 10) for 20 min at 4 °C. Then the slides were washed and immersed in fresh alkaline electrophoresis solution (1 mM EDTA, 300 mM NaOH, pH 13) for 10 min at 4 °C to allow DNA to unwind. Electrophoresis was performed at 300 mA for 10 min. The slides were washed with neutral buffer (0.4 mM Tris–HCl, pH 7.5) and stained with 50 μL propidium iodide solution (PI, 20 μg/mL), images were taken by fluorescence microscopy (Lecois, DM3000, GER). At least 200 cells were analyzed per sample withcomet analysis system (http://www.casp.of.pl).

### DNA double-strand breaks assay

The γ H2AX as a marker of DNA double-strand breaks (DSBs) was performed as described by a previously reported method [20]. QSG7701 cells were treated with 2.5, 5, 10 and 20 μM EMB for 6 h, and 0.1% DMSO was used as the control. Then, cells were fixed in 4% paraformaldehyde for 10 min and permeabilized in 1% Triton-X100 for 10 min. After blocked with 5% BSA for 1 h at room temperature, the cells were incubated with a rabbit monoclonal anti-γH2AX antibody (1:100) overnight in 4°C, and conjugated with Alexa Fluor 488-conjugated anti-rabbit secondary antibody (1:2000) at room temperature for 1 h. For the staining of the nuclei, DAPI (1 mg/mL) was added to the cells and incubated for 10 min at 37 °C in dark. The cells were then mounted in antifade media, and pictures were taken using a confocal laser scanning microscope (Nikon Inc., Melville, N.Y.).

### Chromatin condensation detection

After the QSG7701 cells were treated by specified concentrations of EMB for specified times mentioned in the figure legends, cells were fixed with 4% paraformaldehyde solution for 10 min at 4°C. The fixed cells were washed three times with cold PBS buffer and then stained with 1μL of Hoechst 33258 (1 mg/ml) in 1 mL cold PBS buffer and incubated for 10 min at 37°C in the dark. The images were taken by fluorescence microscopy.

### Apoptosis assay

The Invitrogen™ Alexa Fluor 488 Annexin V/ propidium iodide (PI) assay kit was used to assess apoptosis. After the QSG7701 cells were treated by specified concentrations of EMB for specified times mentioned in the figure legends, cells were collected and washed twice to remove EMB. Then, the cells were labeled with Annexin V-FITC and PI for 15 min at room temperature (25 °C) in the dark. Finally, cells were analyzed by flow cytometer (Becton Dickinson).

### Determination of the mitochondrial membrane potential (*ΔΨm*)

The changes in mitochondrial membrane potential (*ΔΨm*) were determined on the basis of mitochondrial retention of the fluorescent dye Rh123 [21]. A decrease in the fluorescence intensity of Rh-123 indicates a decline in *ΔΨm*. After the QSG7701 cells were treated by specified concentrations of EMB for specified times mentioned in the figure legends, cells were collected and washed twice to remove EMB and stained with Rh-123 at 37 °C for 15 min in the dark. After incubation, the cells were washed twice and. the images were taken by fluorescence microscopy. The fluorescence intensity of 200 cells were analyzed per sample with image analysis system (*ImageJ* software).

### Western blot analysis

After treatment with 2.5, 5, 10 and 20 μM EMB for 6 h, the QSG7701 cells were collected and total protein were extracted in RIPA lysis buffer with PMSF (1mM). Mitochondrial and cytosolic proteins were isolated using the Mitochondria/Cytosol Fractionation Kit. Sample (20-40 μg protein) were separated by electrophoresis on 8–15% SDS-PAGE. Following electrophoresis, the samples were transferred at 20 V for 45 min onto polyvinylidene difluoride (PVDF) membrane. The blots were blocked with 5% BSA and 0.05% Tween 20 in PBS after the transfer for 2 h at room temperature and the PVDF membrane was incubated with primary antibodies overnight at 4°C. Anti-PARP, anti-β-actin monoclonal antibody was diluted 1:1000, anti-cytc monoclonal antibody was diluted 1:200 and anti-Bax, anti-Bcl-2 monoclonal antibody were diluted 1:500 in PBS Tween20 (0.05%, TBST) containing 5% BSA. Then, HRP conjugated secondary antibodies were incubated. Antibodies were detected by a chemiluminescence kit.

### Detection of caspase-3 and caspase-9 activity

The activities of Caspase-3 and Caspase-9 are important markers of apoptosis process in cells. After treatment with 2.5, 5, 10 and 20 μM EMB for 6 h, the QSG7701 cells were collected and washed two times. The activities of caspase-3/-9 were determined using the Caspase-3/-9 activity kit (Beyotime Institute of Biotechnology, Shanghai, China) according to the manufacturer’s instructions. Then, the reading of absorbance was taken at 405 nm by a Synergy H1 microplate reader.

### Intracellular ROS measurement

After treatment with 2.5, 5, 10 and 20 μM EMB for 6 h, the QSG7701 cells were obtained by centrifugation at 100 × g for 5 minutes and washed for two times by cold PBS buffer. Oxidations of DCFH-DA was used to measure ROS produced intracellularly. Then, cells were hatched with 1 mL PBS buffer which contained 10 μM DCFH-DA at 37 °C for 30 minutes allowing fluorescent probe to diffuse into the cells and then hydrolyze to non-fluorescent dichlorofluorescin (DCFH) in response to intracellular esterase. Intracellular generated ROS was observed under fluorescence microscopy with excitation wavelengths fixed at 488 nm and emission wavelengths 528 nm. Fluorescence intensity was analyzed using *ImageJ* software.

### Statistical analysis

Data was presented as the mean ± SD determined from a minimum of three independent experiments. Statistical analysis of the data was performed using one-way analysis of variance (ANOVA) followed by the Dunnet’s test using SPSS 17.0 (SPSS Inc., Chicago, IL, USA). P<0.05 was considered statistically significant.
